# Comprehensive characterization of circulating tumor cells and cell‐free DNA in patients with metastatic melanoma

**DOI:** 10.1002/1878-0261.13650

**Published:** 2024-05-24

**Authors:** Manouk K. Bos, Jaco Kraan, Martijn P. A. Starmans, Jean C. A. Helmijr, Noortje Verschoor, Maja J. A. De Jonge, Arjen Joosse, Astrid A. M. van der Veldt, Peter A. W. te Boekhorst, John W. M. Martens, Stefan Sleijfer, Saskia M. Wilting

**Affiliations:** ^1^ Department of Medical Oncology Erasmus MC Cancer Institute, University Medical Center Rotterdam The Netherlands; ^2^ Department of Radiology and Nuclear Medicine Erasmus MC Rotterdam The Netherlands; ^3^ Department of Hematology Erasmus MC Cancer Institute, University Medical Center Rotterdam The Netherlands

**Keywords:** circulating tumor cells, circulating tumor DNA, liquid biopsy, melanoma

## Abstract

Advances in therapeutic approaches for melanoma urge the need for biomarkers that can identify patients at risk for recurrence and to guide treatment. The potential use of liquid biopsies in identifying biomarkers is increasingly being recognized. Here, we present a head‐to‐head comparison of several techniques to analyze circulating tumor cells (CTCs) and cell‐free DNA (cfDNA) in 20 patients with metastatic melanoma. In this study, we investigated whether diagnostic leukapheresis (DLA) combined with multimarker flow cytometry (FCM) increased the detection of CTCs in blood compared to the CellSearch platform. Additionally, we characterized cfDNA at the level of somatic mutations, extent of aneuploidy and genome‐wide DNA methylation. Both CTCs and cfDNA measures were compared to tumor markers and extracranial tumor burden on radiological imaging. Compared to the CellSearch method applied on peripheral blood, DLA combined with FCM increased the proportion of patients with detectable CTCs from 35% to 70% (*P* = 0.06). However, the median percentage of cells that could be recovered by the DLA procedure was 29%. Alternatively, cfDNA mutation and methylation analysis detected tumor load in the majority of patients (90% and 93% of samples successfully analyzed, respectively). The aneuploidy score was positive in 35% of all patients. From all tumor measurements in blood, lactate dehydrogenase (LDH) levels were significantly correlated to variant allele frequency (*P* = 0.004). Furthermore, the presence of CTCs in DLA was associated with tumor burden (*P* < 0.001), whereas the presence of CTCs in peripheral blood was associated with number of lesions on radiological imaging (*P* < 0.001). In conclusion, DLA tended to increase the proportion of patients with detectable CTCs but was also associated with low recovery. Both cfDNA and CTCs were correlated with clinical parameters such as LDH levels and extracranial tumor burden.

## Graphical abstract

This study provides a comprehensive analysis of liquid biopsies in patients with melanoma, an aggressive form of skin cancer. We profiled cfDNA using these various genetic and epigenetic approaches and we subsequently compared cfDNA to circulating tumor cells with the aim to increase the sensitivity of blood‐based tumor detection.

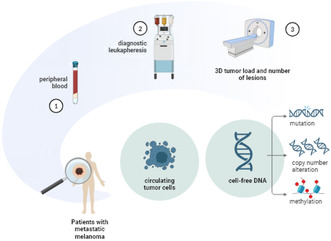

**Abbreviations**
cfDNA: cell‐free DNA
CNA: copy number alteration
cfDNA: cell‐free DNA
ctDNA: circulating tumor DNA
CTC: circulating tumor cell
DMR: differentially methylated region
ddPCR: digital droplet PCR
EDTA: ethylenediaminetetraacetic acid
EPCAM: epithelial cell adhesion molecule
FCM: flow cytometry
FDR: false discovery rate
LOD: limit of detection
MCAM: melanoma cell adhesion molecule
MCSP: melanoma‐associated chondroitin sulfate proteoglycan
NGS: next‐generation sequencing
RBC: red blood cell
VAF: variant allele frequency

## 1 Introduction

The incidence of cutaneous melanoma has been steadily increasing over the past decades [1]. Although the majority of patients can be cured by surgery, 30–70% of patients with stage III disease develop distant metastasis [2]. Currently, the outcome for patients with advanced melanoma has substantially improved with the introduction of novel therapeutic strategies in both the adjuvant and metastatic setting [2, 3]. A selected group of patients with stage IV disease now have durable responses on immunotherapy that can last for years, even after discontinuation of treatment [4]. Although very effective, these treatment regimens are expensive and sometimes very toxic. This underlines the importance of the identification of markers that can serve as a tool to identify patients with disease progression during systemic treatment or patients with early stage melanoma who have with minimal residual disease (MRD) after curative surgery.

Liquid biopsies are gaining ground as a prognostic biomarker in patients with cancer. Also, they have the potential to be used as a predictive marker for treatment outcome. In particular, circulating tumor cells (CTCs) and circulating cell‐free DNA (cfDNA) are promising tools for tumor characterization and monitoring, as both analytes reflect different biological aspects of a tumor [5]. CTCs are intact cells that contain the entire genome of a tumor cell, whereas cfDNA originates from mainly apoptotic cells and is therefore highly fragmented. The main advantage of CTCs is that they can be characterized at the genome, transcriptome and proteome level, including phenotype characterization. In addition, when analyzed as single cells, CTCs can be used to determine the level of intra‐patient tumor heterogeneity [6, 7, 8]. On the contrary, cfDNA represents a mixture of DNA derived from healthy and cancerous cells. The latter is also known as circulating tumor DNA (ctDNA). Whereas cfDNA isolation and characterization is relatively easy, CTC enumeration in patients with melanoma is complicated by the low numbers at which they are present in the circulation [9] and the lack of standardized methods for their isolation and quantification [10]. Techniques to characterize the entire genome and epigenome of cfDNA in a comprehensive and sensitive manner are rapidly progressing. As such, cfDNA analysis has great potential as a readily and easily accessible tool for clinical practice worldwide [11, 12].

The CellSearch system (Menarini Silicon Biosystems, Bologna, Italy) is the only standardized platform that is FDA‐approved for CTC enumeration in breast, colon and prostate cancer [13]. Although this platform benefits from robust, semi‐automated CTC detection, it relies on single marker expression for enrichment of CTCs from the blood. Using this platform, 40% of the patients with metastatic melanoma have detectable CTCs with a median of 2 CTCs per 7.5 mL blood [14]. To increase the limit of detection (LOD) of CTC enumeration, and to increase the amount of cells available for subsequent characterization, the blood collection volume can be increased by using diagnostic leukapheresis (DLA). DLA enables the collection of large numbers of mononuclear cells (MNCs) from the peripheral blood through continuous centrifugation [15]. Since epithelial cells have the same density as MNCs, these cells can effectively be collected during this procedure. Whether this procedure is also effective to collect melanoma CTCs, which are not from epithelial origin, is yet unknown.

In this study, we compared different liquid biopsy assays with the objective to improve the detection and characterization of melanoma contents in blood. As such, we present a head‐to‐head comparison of various tumor‐specific measurements in blood within a cohort of advanced melanoma patients. First, we investigated whether increasing the collection volume by DLA increases the sensitivity of CTC detection when compared to the standard procedure, analysis in 7.5 mL of blood. Also, we investigated whether multimarker flow cytometry (FCM) enabled detection of additional melanoma CTCs when compared to the CellSearch platform as this approach would theoretically enable capture of heterogeneous melanoma cells. Next to CTC analysis, we performed a comprehensive analysis of cfDNA focusing on tumor‐derived mutations, extent of aneuploidy and genome‐wide DNA methylation profiles. Finally, we compared both CTCs and cfDNA to routine markers as LDH and extracranial tumor burden on radiological imaging.

## 2 Materials and methods

### 2.1 Patient population

Between April 2019 and July 2022, we have recruited patients of 18 years of age or older who had metastasis from a histologically confirmed cutaneous melanoma at the Erasmus Medical Center, Rotterdam, The Netherlands. All patients who started with a new line of systemic treatment, irrespective of treatment line, were eligible. As such, all patients had an ECOG performance status of 0–2. Only patients with a known mutation in the tumor based on standard‐of‐care tissue analysis of the primary or metastatic lesion were included. In some cases, only the BRAF gene was analyzed by digital PCR. If the result was negative, tissue DNA was analyzed by an amplicon‐based next‐generation‐sequencing (NGS) panel containing 58 genes selected for clinical relevance (containing at least *CDKN2A*, *PTEN*, *TP53*, *BRAF*, *NRAS*, *MAP2K1*, *ROS1*, *AKT1*, and *TERT* promoter). For this study, patients underwent DLA and peripheral blood collection before the start of systemic treatment. According to standard‐of‐care, serum lactate dehydrogenase (LDH) level was measured (reference < 248 U·L^−1^) and computed tomography (CT) and/or ^15^F‐fluorodeoxyglucose‐positron emission tomography (^15^F‐FDG‐PET) was performed before start of treatment. The study was performed in accordance with the Declaration of Helsinki and approved by the medical ethics committee of the Erasmus MC (MEC18‐1496, Dutch trial register: NTR7557). All patients gave written informed consent before participation.

### 2.2 Assessment of total tumor burden

To correlate the presence of CTCs or ctDNA to tumor burden, we measured the total tumor volume (cm^3^) on the baseline CT‐scan or the low‐dose CT‐scan if only a ^18^F‐FDG‐PET image was available. For all patients, the metastatic lesions were manually segmented (i.e. the tumor lesions were assigned on each image slide) by a clinician on the baseline 3D CT scans using in‐house developed software [16]. All metastatic lesions with a long axis > 10 mm and lymph nodes with a short axis > 15 mm were segmented. Based on the segmentations, the total extracranial tumor burden and the number of lesions were assessed.

### 2.3 Diagnostic leukapheresis and CTC enrichment

Diagnostic leukapheresis (DLA) was performed using the Spectra Optia Cell Separator (Terumo BCT, Lakewood, CO, USA). In total, 5 L of blood were processed per patient for the collection of a mononuclear cell fraction with a density that is similar to CTCs, as previously described for prostate‐derived CTCs [17]. White blood cell (WBC) depletion with RosetteSep™ CTC Human CD45 Depletion Cocktail (STEMCELL Technologies, Vancouver, BC, Canada) was used as described before [17]. Subsequent CTC enumeration in this enriched cell fraction was performed on two platforms: FCM and the CellSearch system. The CTC recovery was calculated as follows: we measured the CTC count in 7.5 mL peripheral blood using the CellSearch platform. We know the total amount of blood processed during the DLA procedure. We could than calculate the amount of CTCs that could theoretically be expected in the total DLA product, based on the total amount of blood processed during DLA. We divided this concentration by the concentration of CTCs that were truly measured in the DLA product by CellSearch to calculate the CTC recovery.

### 2.4 CTC enumeration with flow cytometry

For detection of melanoma CTCs in the WBC depleted fraction using a multimarker analysis, two multi‐color staining panels were designed and tested (Table S1). Both assays contained antibodies against CD45, MCSP (Melanoma‐associated Chondroitin Sulfate Proteoglycan), CD146 and the nuclear dye DRAQ5 as a backbone to identify melanoma CTCs. To explore additional subset(s) of melanoma CTCs, one assay also contained antibodies against CD271, CD274, DAPI as a vitality dye and the other antibodies against the intracellular epitopes GP100 and MART‐1. Cells in second assay were fixed and permeabilized using the FIX&PERM® Kit (Caltag Medsystems, Buckingham, UK) prior to staining for both membrane and intracellular epitopes. After incubation for 15 min at room temperature in the dark, cells were washed with 10 mL PBS. After centrifugation (8 min at 600 ***g***), cell pellets were collected in 500 μL PBS and the entire cell pellet was analyzed on a LSR Fortessa flow cytometer (BD Biosciences, NJ, USA). FCM data was analyzed using fcs express Software (De Novo Software, Pasadena, CA, USA). To determine the optimal dilution, the antibodies were titrated on melanoma cell lines positive for one or more markers (SKMEL‐28, purchased from ATCC and MEL2A, provided by the tumor immunology laboratory of the Erasmus MC), whilst leukocytes from healthy blood donors (HBDs) were used as negative controls. To stimulate CD271 expression, the cell line MEL2A was incubated with interferon‐γ at a concentration of 1000 IU·mL^−1^ for 72 h before harvesting and subsequent titration. Specificity of the FCM antibody conjugate panel for melanoma CTCs was demonstrated by analysis of an aspirate from a metastasectomy specimen of a cystic melanoma metastasis, which contained high purity of tumor cells (Fig. S1). Subsequent mutation analysis was performed on these cells, as outlined in the first paragraph of the results. DNA was isolated from the cell pellet using the NucleoSpin (Macherey‐Nagel, Düren, Germany) and subsequently used to verify the presence of the somatic *BRAF* p.V600E mutation that was identified in tumor tissue from the same patient used for inclusion.

### 2.5 Cell lines

SK‐MEL‐28 (RRID:CVCL_0526, purchased from ATCC, VA, USA), and Mel‐2a (RRID:CVCL_A759, provided by the tumor immunology laboratory of the Erasmus MC) were cultured in RPMI 1640 medium (Thermo Fisher Scientific, Waltham, MA, USA) containing 10% FBS (Lonza, Walkersville, MD, USA) and penicillin/streptomycin (Thermo Fisher Scientific) at 37 °C and 5% CO_2_. Cell lines were authenticated by Short Tandem Repeat profiling (STR) and checked for the absence of mycoplasma contamination using the MycoAlert assay (Lonza). The PowerPlex 16 System (Promega, Madison, WI, USA) was used according to the manufacturer's protocol using an ABI PRISM 3100 to generate an STR (Short tandem repeat) fingerprint of each cell line to determine unique identity and/or lack of co‐culture contamination. In addition, the STR profile was checked before and during culturing of the cells to ensure that cross‐contamination or erroneous substitution did not occur.

### 2.6 CTC enumeration with CellSearch

CTCs were enumerated using the CellSeach assay as described before [9]. In short, blood samples (7.5 mL each) and enriched DLA samples (1–2 mL each, depending on the leukocyte concentration as a maximum of 200 × 10^6^ leukocytes could be analyzed by the CellSearch platform) were collected in CellSave tubes (Menarini Silicon Biosystems). The circulating melanoma cell enumeration kit (Menarini Silicon Biosystems) was applied for CTC enrichment and enumeration. This assay enriches melanoma cells using MCAM and subsequently uses a combination of MCSP and nuclear dye to identify melanoma CTCs.

### 2.7 Cell‐free DNA isolation

Plasma from EDTA‐containing tubes was separated within 2 h after collection and isolated using two sequential centrifugation steps [18]. Subsequently, cfDNA was isolated from four milliliters of plasma using the QIAamp Circulating Nucleic Acids kit (Qiagen, Venlo, The Netherlands). cfDNA concentrations were measured using the Quant‐iT dsDNA high‐sensitivity assay (Invitrogen, Life Technologies, Carlsbad, CA, USA) on the Qubit fluorometer (Invitrogen) according to the manufacturer's instructions [18].

### 2.8 cfDNA mutation analysis

Digital droplet PCR (ddPCR) was used to evaluate the presence of clonal mutations that were found in primary or metastatic tumor tissue in cfDNA as well as sorted CTCs. For cfDNA, a volume of 10 μL of cfDNA eluate was used as input. For sorted melanoma cells and lymphocytes, DNA was pre‐amplified before analyses using the Tagman™ PreAmp Master Mix. Analysis of mutations was performed using uniplex ddPCR mutation assays (*BRAF* p.V600E, *NRAS* p.Q61K/R, *BRAF* p.N581S, *TERT* C228T/C250T) from Bio‐Rad Laboratories or ThermoFisher as previously described [19] on the Naica System (STILLA Technologies, Villejuf, France). For *TERT* promoter 228T/250T mutations, a different PCR master mix (Master Mix for PCR, Bio‐Rad, San Francisco, CA, USA) was used to improve the signal‐to‐noise ratio in the blue channel.

### 2.9 cfDNA aneuploidy analysis

We estimated the ctDNA fraction within the total pool of cfDNA using the modified fast aneuploidy screening test‐sequencing system (mFast‐SeqS) as described before [20]. This method amplifies and sequences LINE‐1 elements, which are short fragments distributed throughout the genome, enabling aneuploidy analysis of cfDNA [21]. Briefly, a z‐score per chromosome arm was calculated by subtracting the mean and dividing by the SD of normalized read counts for the respective chromosome arm from a panel of HBDs. We ensured that all samples had at least 90 000 mapped reads, which was previously shown to be sufficient for analysis [20]. Finally, Z‐scores per chromosome were squared and summed into a genome‐wide aneuploidy score per patient.

### 2.10 cfDNA methylation analysis

We obtained genome‐wide cfDNA methylation profiles using the MeD‐seq method [11], which uses LpnPI methylation‐dependent restriction enzyme to select methylated DNA sequences for high throughput NGS. The workflow for data processing, analysis and statistical testing was previously described [11]. In short, only regions containing data in at least 75% of all samples and nine HBD controls [11] were included in the analysis, resulting in a total of 38 610 regions on chromosome 1–22. Principal component analysis was performed on the 50% most variable regions. To generate one overall score for aberrant cfDNA methylation per patient, z‐scores were calculated per region, normalizing for scores from this panel of nine HBDs. These Z‐scores per region were squared and summed into a genome‐wide Z‐score per patient. To further specify this score for melanoma‐specific reads, this was repeated using only data from 118 regions that were differentially methylated (FDR < 0.1) according to LIMMA analysis [22] between HBDs and patients with a high fraction of ctDNA (defined as an mFast‐SeqS Z‐score ≥ 3). To set a cut‐off for patients with an altered overall methylation profile, we took the upper limit of the 95% confidence interval (CI) of the genome‐wide z‐scores from nine HBDs.

### 2.11 Statistical analysis

Other studies have shown that CMCs detected in 7.5 mL of peripheral blood by CellSearch method are found in 40% of patients with metastatic melanoma. We consider a putative CMC detection rate by flow cytometry of 70% in metastatic melanoma suitable for further exploration of this technique in the adjuvant setting. Therefore, we applied an optimal Simon two‐stage design with p0 = 0.4 and p1 = 0.7, α = 0.05 and β = 0.2, resulting in a sample size of 20 patients. Mann–Whitney *U* test was performed for univariate analyses of continuous variables and a Fisher exact test was used for categorical variables. To calculate the correlation between LDH or tumor volume and CTC count or ctDNA fraction, we calculated the Spearman ρ. Statistical tests were two‐sided and considered statistically significant when *P* < 0.05. IBM SPSS STATISTICS 25 (ICM Corp, Armonk, NY, USA) and Prism™ software (GraphPad Software, La Jola, CA, USA) was used for the statistical analyses.

## 3 Results

### 3.1 Patient population

In total, 20 patients were included in this analysis. Of those, 65% (*n* = 13/20) had either a *BRAF* V600E or V600K mutation, 15% (*n* = 3/20) had another *BRAF* mutation (p.K601N and p.N581S) and 20% (*n* = 4/20) had an *NRAS* mutation (Table 1). Most patients had not received any systemic treatment for metastatic disease (85%, *n* = 17/20) and 40% (*n* = 8/20) had brain metastases. Of all patients, 35% (*n* = 7/20) had detectable melanoma CTCs in 7.5 mL of blood using the CellSearch platform whereas 90% (*n* = 18/20) had a detectable somatic mutation in cfDNA with a median variant allele frequency (VAF) of 3.2% (range: 0.2–30.7%).

**Table 1:** Overview of clinical characteristics, circulating tumor cell counts and circulating tumor DNA fractions on all patients included in this study.

Characteristics	Total (*N* = 20)
Age (years), Median (range)	57 (29–76)
Gender	
Male, *n* (%)	13 (65)
Female, *n* (%)	7 (35)
Genetic subtype	
*BRAF* p.V600E/K mutation, *n* (%)	13 (65)
*NRAS* mutation, *n* (%)	4 (20)
Other mutation, *n* (%)	3 (15)
Prior treatment for metastatic disease	
Yes, *n* (%)	3 (15)
No, *n* (%)	17 (85)
Brain metastasis	
Yes, *n* (%)	8 (40)
No, *n* (%)	12 (60)
Extracranial tumor load (cm^3^), Median (range)	107 (2–736)
LDH (U·L^−1^), Median (range)	247 (164–1074)
Detectable CTCs in 7.5 mL of peripheral blood by CellSearch	
Yes, *n* (%)	7 (35)
No, *n* (%)	13 (65)
ctDNA variant allele frequency of dominant mutation (%), Median (range)	3.18 (0–55.8)

### 3.2 Diagnostic leukapheresis increased the number of patients with CTCs but melanoma CTCs were present in low numbers

**Fig. 1:**
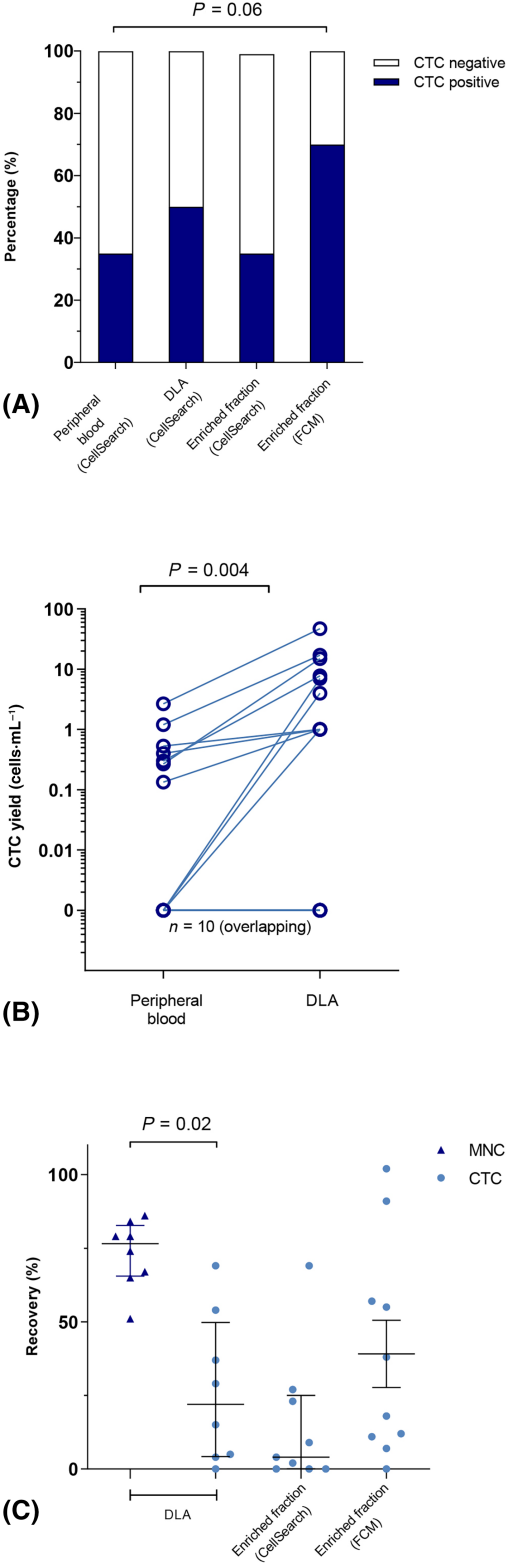
Efficiency of circulating tumor cell (CTC) enrichment and isolation techniques from diagnostic leukapheresis. (A) Percentage of patients with detectable CTCs using different enumeration platforms. Statistical comparison was performed using a Fishers' exact test. (B) CellSearch CTC yield in peripheral blood (PB) and diagnostic leukapheresis (DLA) product in all patients. The *y*‐axis shows CTC count per mL PB and per mL DLA product. Each dot represents an individual subject (*n* = 15, flat line are 10 patients). Statistical comparison was performed using a paired two‐sided Wilcoxon rank test. (C) Relative CTC recovery (%) and mononuclear cell recovery (%) after DLA and white blood cell (WBC) depletion in CTC positive patients. To calculate CTC recovery, the estimated CTC count after the apheresis/enrichment was divided by the estimated CTC count before the apheresis/enrichment. Mononuclear cells include lymphocytes and monocytes and recovery was calculated similar to CTCs. Lines indicate the median and interquartile range. Statistical comparison was performed using a paired two‐sided Wilcoxon rank test.

The median blood volume that was processed during the apheresis procedure was 5.0 L (range: 2.4–6.8 L) of blood and the median product volume after apheresis was 93 mL (range: 46–121 mL) with a median white blood cell count (WBC) of 114 × 10^9^·L^−1^ (range: 49–244 × 10^9^·L^−1^). No patients experienced adverse events during the diagnostic apheresis procedure. Using DLA combined with subsequent CTC enrichment, the percentage of patients with detectable CTCs tended to increase from 35% to 50% when analyzed by CellSearch and ultimately to 70% by FCM (*P* = 0.06, Fig. 1A). DLA increased the median CTC count from 0 cells·mL^−1^ in peripheral blood to 1 cells·mL^−1^ in the DLA product, using CellSearch platform, *P* = 0.004 (Fig. 1B). Although we increased the number of patients with detectable CTCs by DLA, the recovery of CTCs was only 29% whereas for MNCs, this median fraction was 70% (*P* = 0.02, Fig. 1C). After CTC enrichment, the median recovery was 28% (range: 0–102%) with FCM whereas with CellSearch the median recovery was 4% (range: 0–69%). In patients in whom ≥ 5 MCAM^+^/MCSP^+^ melanoma CTCs were detected, no substantial heterogeneity was observed among the main melanoma‐specific membrane and intracellular markers (MCAM, MCSP, gp100 and Melan‐A, Fig. S2, Table S2). In one patient (subject 13), > 100 putative melanoma‐derived CTCs were identified in the DLA product, allowing us to isolate these cells by fluorescence‐activated cell sorting (FACS) using a FACSAria cell sorter (BD BioSciences). Cells, both putative CTCs and lymphocytes, were sorted in 300 μL of PBS, centrifuged at 12 000 ***g*** for 5 min and snap‐frozen at −80 °C until further processing. In this patient the *BRAF* V600E mutation was identified in sorted CD146^+^/MCAM^+^/CD45^−^ cells, whilst the mutation was not identified in sorted leukocytes (MCAM^−^/MCSP^−^/CD45^+^ cells) from the same specimen (Fig. S3), confirming that MCAM^+^/MCSP^+^ cells identified by FCM indeed represent true melanoma cells.

### 3.3 Comprehensive cfDNA analysis

**Fig. 2:**
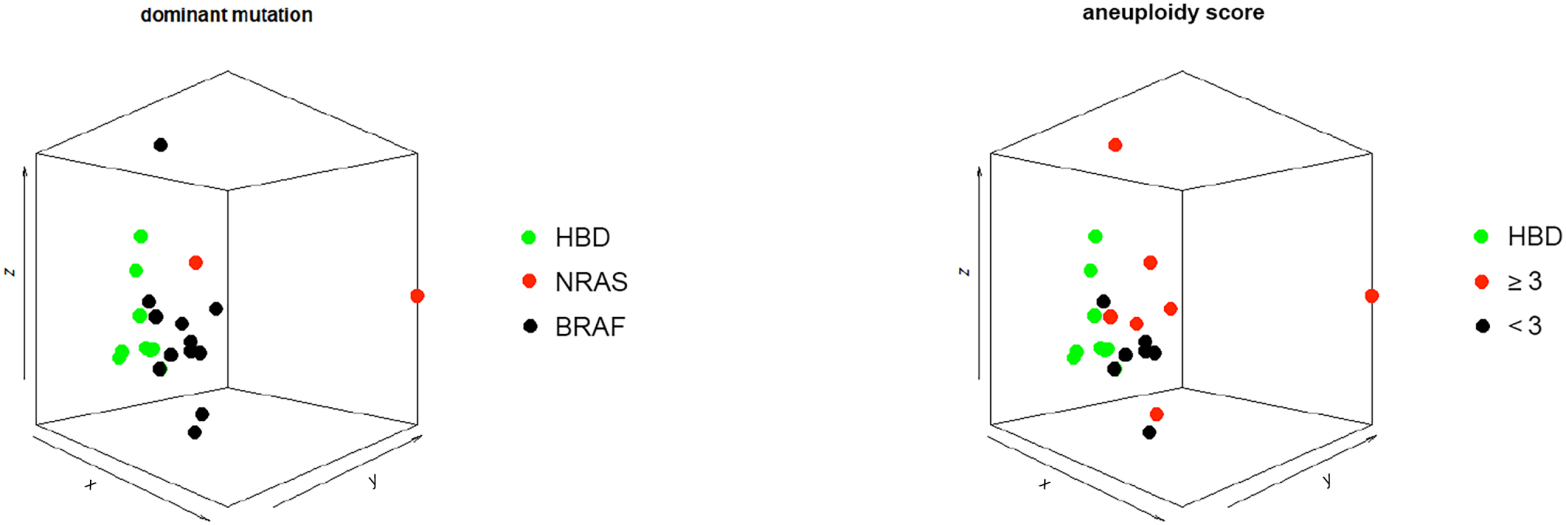
Principal component analysis of MeD‐seq cell‐free DNA (cfDNA) methylation profiles from healthy blood donors (HBDs) and patients included in the study. PC1, PC2, and PC3 are shown on the *x*‐axis, *y*‐axis and *z*‐axis respectively. Left: samples are colored based on their genetic subtype. Right: samples are colored based on their aneuploidy score (fast‐seq).

We further characterized cfDNA from all patients in our study at the level of somatic mutations, genome‐wide aneuploidy score and genome‐wide methylation. Of the 18 patients in whom a dominant mutation was detectable in cfDNA, 9/18 (50%) mutations had VAF ≥ 5%. In total, seven patients (35%) had a aneuploidy score of ≥ 3. The VAF was correlated with the genome‐wide aneuploidy score (Spearman's ρ 0.720, *P* ≤ 0.001, Fig. S4). In nine patients with a VAF ≥ 5%, 7 (77%) patients had an aneuploidy score ≥ 3. Remarkably, three out of four patients with a *NRAS* mutation (75%) had an aneuploidy score of ≥ 20. Although those patients also had a VAF ≥ 25%, we did not observe such high aneuploidy scores nor similarly high VAFs in a patients having a *BRAF* mutation. cfDNA methylation data were successfully generated in 14 out of 20 patients (70%). Principal component analysis on the 50% most variable regions of all genome‐wide methylation profiles showed that some patients clustered together with HBDs, which might suggest that that no tumor fraction could be identified by methylation analysis in these patients. To further investigate this, principal component analysis are also presented stratified by aneuploidy score ≤ 3 (Fig. 2), ≥ 1 detectable CTC or VAF ≥ 1% (Fig. S5). When compared to HBDs, 118 DMRs (FDR < 0.1) were identified in all patients with an aneuploidy score ≥ 3 (Table S3). Based on these regions we calculated the melanoma‐specific methylation score for all patients and HBDs. In patients, this melanoma‐specific methylation score showed a significant correlation to the VAF of somatic mutations (Spearman's ρ 0.805, *P* ≤ 0.001, Fig. S4) and the aneuploidy score (Spearman's ρ 0.930, *P* ≤ 0.001, Fig. S4). In general, the number of patients with an altered melanoma‐specific methylation score (defined as a score higher than the upper limit of the 95% confidence interval of the DMR z‐score on HBDs) was 93% (*n* = 13/14). All individual cfDNA measurement data are summarized in Table S4.

### 3.4 Associations between tumor‐specific measurements in the blood and clinical parameters

**Fig. 3:**
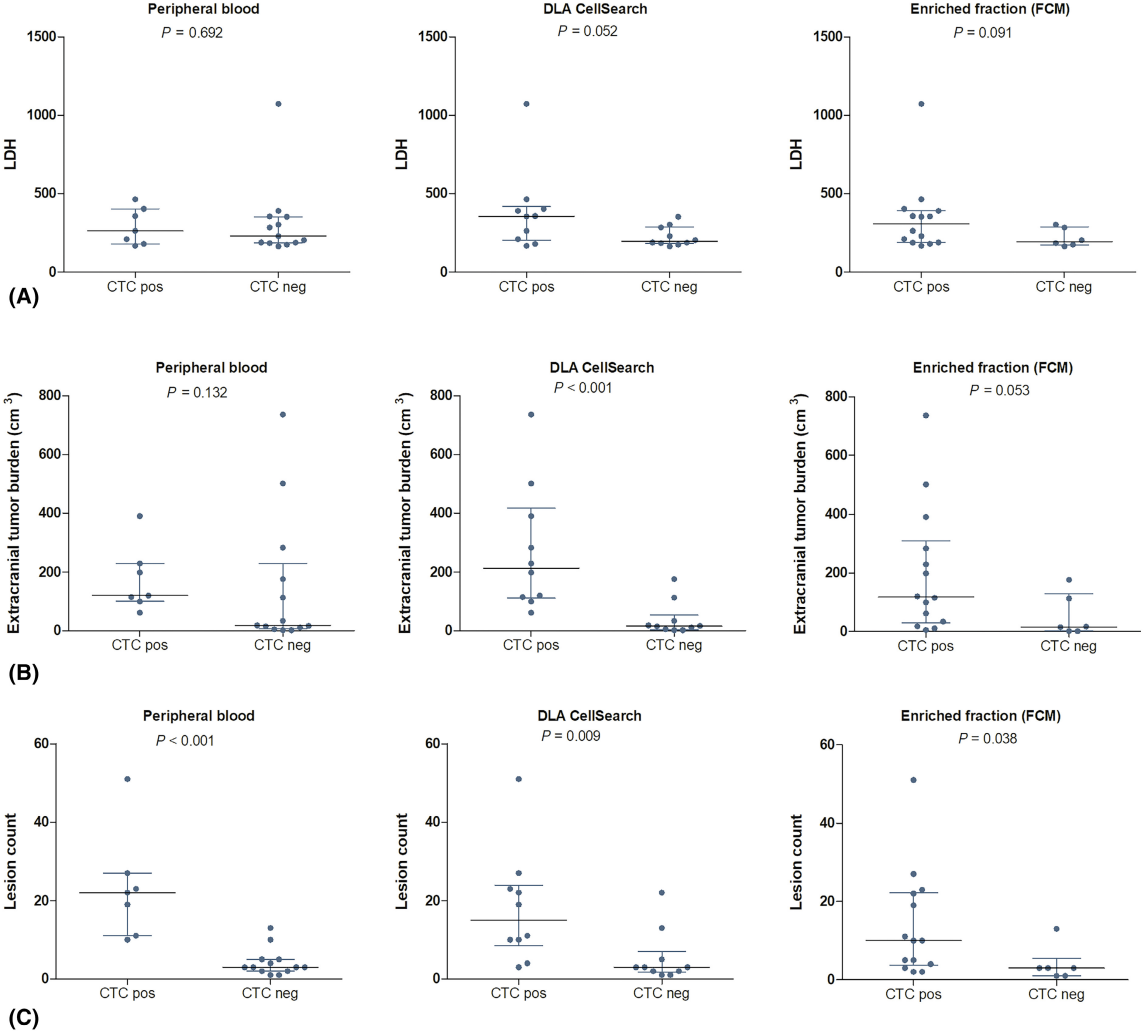
Correlation of circulating tumor cell (CTC) count to other clinical parameters. (A) Mean lactate dehydrogenase (LDH, U·L^−1^) among CTC positive and CTC negative patients on different CTC platforms. (B) Mean extracranial tumor burden (cm^3^) among CTC positive and CTC negative patients on different CTC platforms. (C) Mean lesion count among CTC positive and CTC negative patients on different CTC platforms. Differences were tested using a Mann–Whitney *U* test. Lines indicate the median and interquartile range.

**Fig. 4:**
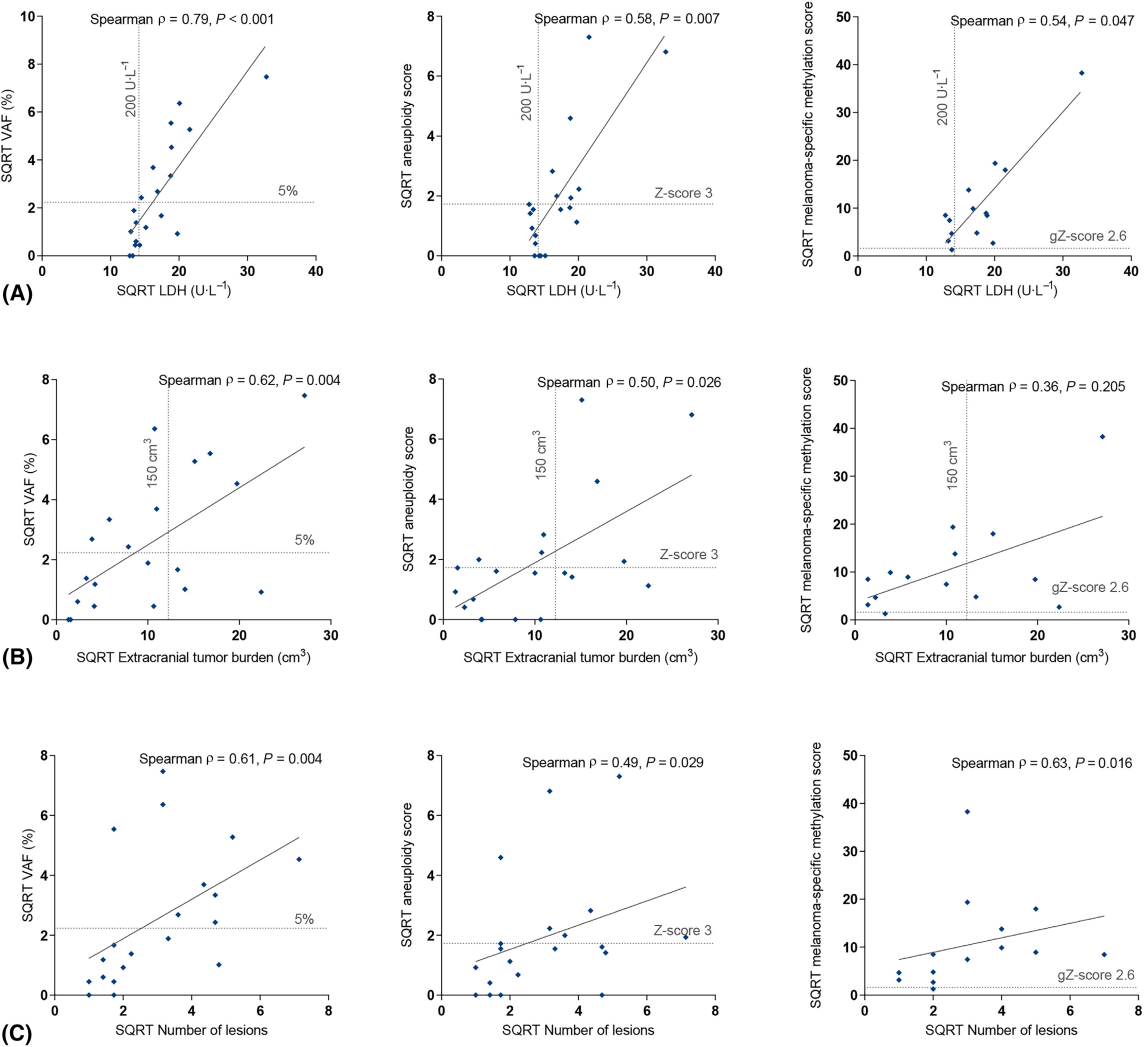
Integrative analysis of cell‐free DNA (cfDNA) analysis methods and the correlation to other clinical parameters. (A) Correlations between lactate dehydrogenase (LDH, U·L^−1^) and variant allele frequency (VAF), mFast‐Seqs Z‐score and MeD‐seq differentially methylated regions (DMR) Z‐score. (B) Correlations between extracranial tumor burden (cm^3^) and VAF, mFast‐Seqs Z‐score and MeD‐seq DMR Z‐score. (C) Correlations between lesion count and VAF, mFast‐Seqs Z‐score and MeD‐seq DMR Z‐score. Spearman's rank correlation was used to test the correlations. To reduce skewness in the data distribution, the square roots (SQRT) were plotted.

We subsequently correlated CTC and cfDNA measurements with LDH levels, tumor burden and the number of lesions on the CT‐scan. For CTC measurements, LDH levels were not significantly different between patients with or without detectable CTCs on different platforms (Fig. 3A). In cfDNA, the VAF of a somatic mutation was strongly correlated with serum LDH levels (Spearman's ρ = 0.79, *P* < 0.001, Fig. 4A).

The extracranial tumor burden was higher in patients with detectable CTCs in DLA material using CellSearch than in patients without detectable CTCs (213 vs. 16 cm^3^, *P* < 0.001, Fig. 3B). In cfDNA, the correlations between different cfDNA platforms and extracranial tumor burden were less strong, although the correlation between the VAF and the extracranial tumor burden was significant (Spearman's ρ = 0.62, *P* = 0.004, Fig. 4B). There was no difference in CTC and cfDNA measurements between patients with or without metastases to the liver.

The number of lesions on the CT‐scan were higher among patients with detectable CTCs on all platforms than in patients without detectable CTCs on all platforms (Fig. 3C). In cfDNA, only the VAF was somewhat correlated with the number of lesions (Spearman's ρ = 0.61, *P* = 0.004, Fig. 4C).

### 3.5 Different liquid biopsy assays to detect disease in blood of melanoma patients

**Fig. 5:**
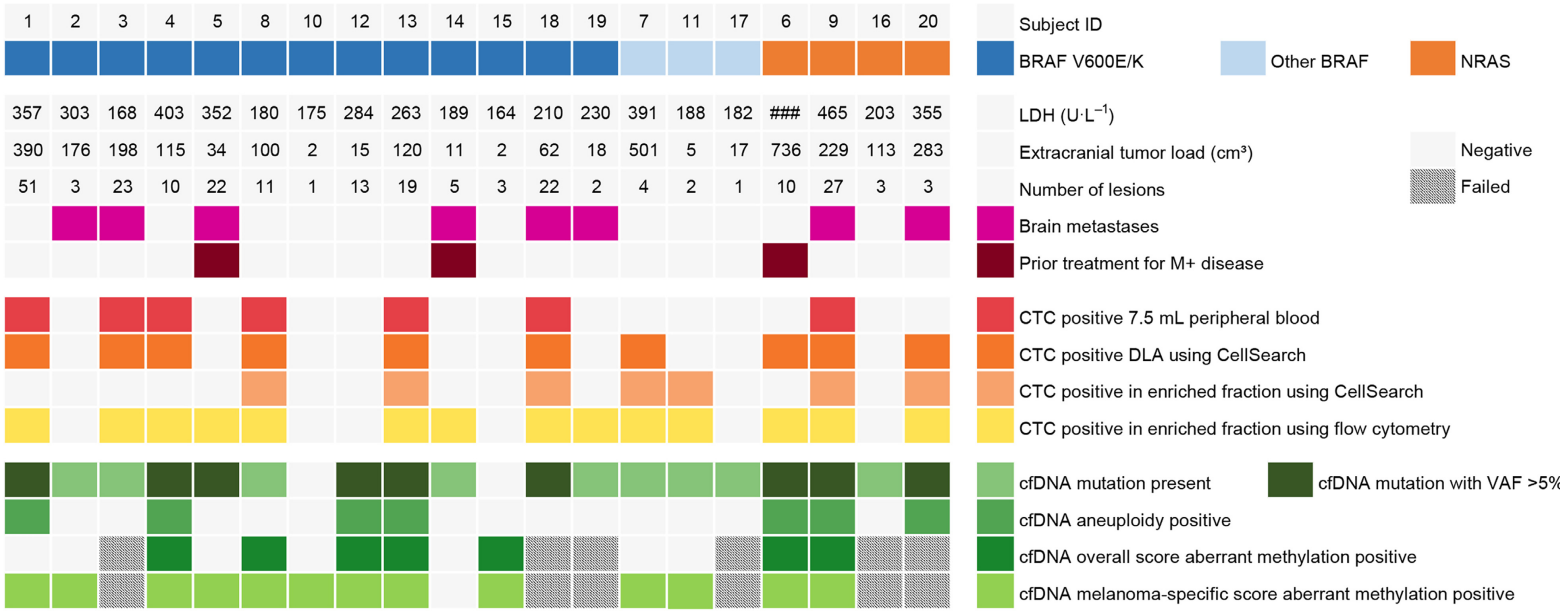
Comprehensive overview of clinical characteristics, circulating tumor cell (CTC) count and cell‐free DNA (cfDNA) characteristics of patients included in this study. For the MeD‐seq genome‐wide Z‐score and the MeD‐seq differentially methylated regions (DMR) z‐score, the upper limit of the 95% confidence interval based on the healthy donor profiles were chosen as a cut‐off.

Although the primary aim of this study was not to compare different liquid biopsy assays in patients with metastatic melanoma, Fig. 5 provides a summary of the clinical characteristics, CTC counts and cfDNA characteristics of the patients included in this study. In our cohort, the number of patients with a detectable mutation in cfDNA was higher compared to the number of patients with detectable CTCs in peripheral blood (90% or 18/20 vs. 35% or 7/20, *P* < 0.001) or DLA (90% vs. 50%, *P* = 0.014). Although FCM analysis identified more patients with detectable CTCs (14/20) than the CellSearch platform (7/20), all patients with detectable CTCs using FCM also had a detectable mutation in cfDNA. Interestingly, two patients (subject 10 and 15) in whom neither CTCs nor a mutation was detected, the melanoma‐specific methylation score was altered.

## 4 Discussion

In this study, we compared different CTC and cfDNA detection methods with the aim to improve the detection and characterization of melanoma contents in blood. Therefore, patients with metastatic melanoma who started a new line of treatment underwent peripheral blood collection and DLA for enrichment of CTCs. We demonstrated that DLA tended to increase the proportion of patients with detectable CTCs but was also associated with low recovery. Contrarily, cfDNA mutation and methylation analyses detected circulating tumor material in the vast majority of patients. Both cfDNA and CTCs were correlated with clinical characteristics such as lactate dehydrogenase levels and extracranial tumor burden. Because of the limited sample size of this study, we did not perform any correlation with clinical outcome. We demonstrated that DLA tended to increase the detection rate of CTCs compared to analysis of CTCs in peripheral blood. Especially by using DLA combined with FCM, we identified melanoma CTCs in more than half of all patients. Unlike other studies that used marker‐dependent approaches including FACS analysis or microfluidic devices [23, 24, 25] to identify melanoma CTCs, we did not observe substantial heterogeneity among the majority of melanoma markers. Our results indicate that single marker CTC capture methods might be sufficient. Contrarily, studies that used marker‐independent enrichment techniques [24] suggest that different phenotypes exist, possibly driven by the molecular make‐up of the cells. As a complicating factor, true melanoma origin has not been formerly established in these studies, rendering the identification of different phenotypes difficult.

This study is the first to demonstrate the true melanoma origin of CTCs identified by FCM, by performing molecular analysis after cell sorting on a sample with a high CTC countTherefore, we confirmed that our FCM assay is specific for melanoma CTCs. Nevertheless, melanoma CTCs were present at a low frequency. Moreover, the recovery of melanoma CTCs using DLA was substantially decreased (29%) when compared to MNCs (70%), indicating that DLA will not be the optimal approach for enrichment of melanoma CTCs. In our experience with DLA in patients with metastatic prostate cancer, the median recovery of CTCs from DLA was 36% of the estimated CTCs present given the processed blood volume and the CTC count in PB [17]. In contrast to CTCs from epithelial origin, melanoma CTCs might be more fragile and, as a result, more susceptible for loss during apheresis or subsequent depletion procedures. Also, we observed that in the enriched fraction, we identified more CTCs by using flow cytometry than by using CellSearch. This might be explained by the fact that the CellSearch platform is a semi‐automated platform which might result in loss of more cells. Alternatively, another possible explanation might be that the FITC channel of the CellSearch platform is less sensitive than the one on the flow cytometer. In all, this raises the question whether CTC enumeration and characterization using this DLA technique in patients with melanoma is worth further exploration, especially since the added value of DLA mainly resides in the collection of larger numbers of CTCs in a research setting, enabling transcriptional or molecular analysis of CTCs [26].

Contrarily, isolation and characterization of cfDNA is relatively easy and ctDNA was detected in most patients in this study (90% with mutation analysis). We compared mutation analysis by ddPCR to mFast‐SeqS, which is an affordable technique to identify tumor load in blood by assessing CNAs and does not require prior knowledge on a patients' mutation status. Although the majority of melanomas are characterized by a clonal mutation in either *BRAF* or *NRAS*, likely limiting the added clinical value of mFast‐SeqS in patients with melanoma, we demonstrated that ddPCR showed a high correlation with mFast‐SeqS. Whereas previous studies have established a genome‐wide z‐score of ≥ 5 to select plasma samples with high tumor fractions [21], we observed a high concordance between a VAF ≥ 5% and a mFast‐SeqS z‐score of ≥ 3 in our cohort.

We further characterized genome‐wide cfDNA methylation patterns using a novel method that is not dependent on bisulfite conversion or antibody‐binding affinity enrichment. When restricting the summary z‐score to the most differential methylated regions for melanoma, we found altered methylation scores in patients with a VAF as low as 1%, suggesting that methylation profiles can be obtained in patients with a generally low circulating tumor fraction. However, methylation analysis failed in 30% of all samples. This was caused by failed LpnPI digestion (i.e. less than 20% of reads passed the LpnPI filter in the first 2M reads). Unfortunately, methylation analysis could not be repeated due to an insufficient amount of cfDNA, In our experience this is an exceptionally high failure rate. Similar to the mFast‐SeqS z‐score, methylation profiles of *NRAS* mutated patients also appeared different from both HBDs and *BRAF* mutated patients, although this study included only a limited number of patients with a *NRAS* mutation. Differential methylation in *NRAS* mutated melanomas, especially promoter hypermethylation of p16, was previously observed [27]. A majority of patients with melanoma have a driving mutation that could be detected in plasma for disease monitoring. However, DNA methylation is also a known factor driving disease progression and, more importantly, resistance to treatment [27, 28]. As such, methylation analysis of cfDNA might be useful for early detection of resistance to treatment or discovery of new resistance mechanisms.

Other studies have previously investigated the value of cfDNA analysis in patients with melanoma [29, 30, 31]. Those studies mainly demonstrate the prognostic value of the presence of somatic mutations in cfDNA in both stage III and IV disease. Also, this prognostic value was shown to be independent from serum LDH levels [30]. As such, cfDNA poses a promising marker to aid clinical management of patients with melanoma, especially in a context in which novel therapeutic regimens have developed quickly. The results of our study underline the feasibility of somatic mutation detection in plasma as it detects tumor load in the majority of patients and is a readily and easily accessible tool. Alternatively, we demonstrate that methylation analysis is equally likely to detect tumor load in blood than somatic mutation analysis.

## 5 Conclusion

In conclusion, this study aimed to improve detection of melanoma tumor load in blood. We demonstrated that using a analysis pipeline with flow cytometry increased the number of patients with detectable circulating tumor cells. However, pre‐analytical steps of this pipeline yielded low recovery. Furthermore, the results of this study suggest that cfDNA assays such as ddPCR and MeD‐seq, the latter which was used to derive a melanoma‐specific methylation score, detected tumor content more frequently than any of CTC assays we tested here. Together with the potential added prognostic value of cfDNA to other markers like serum LDH [32], this should be investigated in a larger patient cohort in future research. Moreover, our results suggest that the phenotype of melanoma CTCs, based on our marker panel, might be less heterogenic than previously thought, although the absolute number of detected CTCs remained low irrespective of the method used. These low number of CTCs and the disappointing recovery rates when using DLA to increase the number of CTCs may limit the use of this workflow to investigate genetic heterogeneity on single CTCs. Alternatively, cfDNA mutation and methylation analysis detected tumor load in the majority of patients. However, cfDNA methylation analysis using MeD‐seq yielded a high failure rate as this technique is relatively new. Whereas cfDNA mutation analysis using ddPCR is a low‐cost assay to estimate tumor fractions in blood by measuring a genetic alteration, cfDNA methylation analysis might provide additional predictive or prognostic information which should be confirmed in future studies.

## Conflict of interest

MKB: Research funding: Dutch Cancer Society (no. NKB‐EMCR‐2016‐108 154). AAMV: advisory board (all paid to institution) of BMS, MSD, Merck, Pfizer, Ipsen, Eisai, Pierre Fabre, Roche, Novartis, Sanofi, all outside the submitted work. JWMM: Sponsorship for research: Pfizer, Menarini, GSK Cytotrack, Cergentis, Payment for consultancy: Novartis, Payment for presentation: Roche, all outside the submitted work. The other authors declare no conflict of interest.

## Author contributions

MKB, NV, MJAJ, AJ, AAMV and PAWB were involved in execution of the study and patient inclusion. MKB, JK, JH, NV, and SMW were responsible for the laboratory analyses and procedures. JWMM, SS and SMW were involved in the conceptualization of the study. MPAS performed the imaging segmentation analyses. All authors were involved in data interpretation. MKB wrote the first draft of the manuscript which was reviewed and edited by all authors. All authors have read and agreed to the published version of the manuscript.

### Peer review

The peer review history for this article is available at https://www.webofscience.com/api/gateway/wos/peer‐review/10.1002/1878‐0261.13650.

## Supporting information


**Fig. S1.** Flow cytometry analysis showing the specificity of the antibody conjugate panel for melanoma CTCs, demonstrated on an aspirate from a metastasectomy specimen of a cystic melanoma metastasis, which contained high purity of tumor cells.
**Fig. S2.** Overview of the flow cytometry analysis of patients with more than five MSCP^+^/CD146^+^ events using the gating strategy.
**Fig. S3.** Digital droplet PCR for *BRAF* V600K mutation on sorted cells from subject 13 using fluorescence‐activated cell sorting (FACS).
**Fig. S4.** Correlation of cell‐free DNA (cfDNA) measurement platforms.
**Fig. S5.** Principal component analysis of MeD‐seq cfDNA methylation profiles from healthy blood donors (HBDs) and patients included in the study.
**Table S1.** Multi‐color staining panel, using two tubes for membrane and intracellular markers.
**Table S2.** Overview of melanoma markers in all subjects.
**Table S3.** List with 118 differentially methylated regions in patients with a mFastSeqS z‐score ≥ 3 and false discovery rate < 0.1.
**Table S4.** Variant allele frequency, aneuploidy score and melanoma‐specific methylation score measurements for each individual patient.

## Data Availability

The datasets used and/or analyzed during the current study are available from the corresponding author on reasonable request.
