# Supplementary material for: Comprehensive characterization of circulating tumor cells and cell‐free DNA in patients with metastatic melanoma
Source: Mol Oncol. 2024 May 24;18(11):2770–82. doi: 10.1002/1878-0261.13650 (PMC11547238; doi:10.1002/1878-0261.13650)
Supplement: Supplementary file 1 — Fig. S1. Flow cytometry analysis showing the specificity of the antibody conjugate panel for melanoma CTCs, demonstrated on an aspirate from a metastasectomy specimen of a cystic melanoma metastasis, which contained high purity of tumor cells. Fig. S2. Overview of the flow cytometry analysis of patients with more than five MSCP+/CD146+ events using the gating strategy. Fig. S3. Digital droplet PCR for BRAF V600K mutation on sorted cells from subject 13 using fluorescence‐activated cell sorting (FACS). Fig. S4. Correlation of cell‐free DNA (cfDNA) measurement platforms. Fig. S5. Principal component analysis of MeD‐seq cfDNA methylation profiles from healthy blood donors (HBDs) and patients included in the study. Table S1. Multi‐color staining panel, using two tubes for membrane and intracellular markers. Table S2. Overview of melanoma markers in all subjects. Table S3. List with 118 differentially methylated regions in patients with a mFastSeqS z‐score ≥ 3 and false discovery rate < 0.1. Table S4. Variant allele frequency, aneuploidy score and melanoma‐specific methylation score measurements for each individual patient. [file MOL2-18-2770-s001.docx]

**Supplemental figures**

**Figure S1.** Flow cytometry analysis showing the specificity of the antibody conjugate panel for melanoma CTCs, demonstrated on an aspirate from a metastasectomy specimen of a cystic melanoma metastasis, which contained high purity of tumor cells. Density plots: **A**. selection of nucleated cells based on DNA content using DRAQ5. **B**. Excluding unspecific events using a negative gate at the empty PE-Texas Red channel. **C**. Composition of the nucleated cells, showing Melanoma Cells(blue circle; CD45^low^,SCC^High^), Granulocytes(CD45^dim^,SCC^High^), Monocytes(CD45^high^,SCC^intermediate^) and Lymphocytes(CD45^high^,SCC^low^). **D.** Gating in CD146^+^ melanoma cells(22,5%) **E-I**. Expression of melanoma markers, respectively MCSP, CD271, CD274 GP100 and Melan-A on CD146^+^ melanoma cells.

A B C

G H I

D E F

**Figure S2.** Overview of the flow cytometry analysis of patients with more than 5 MSCP+/CD146+ events using the gating strategy as described and shown in figure S1 ( Row 1). Row 2-5 are overlay histograms combining Lymphocytes(serving as an internal control, green dots) and melanoma CTCs(purple dots). Melanoma markers, respectively CD146, CD271, CD274 GP100 and Melan-A are plotted on the X-axis against MSCP on the Y-axis.

**
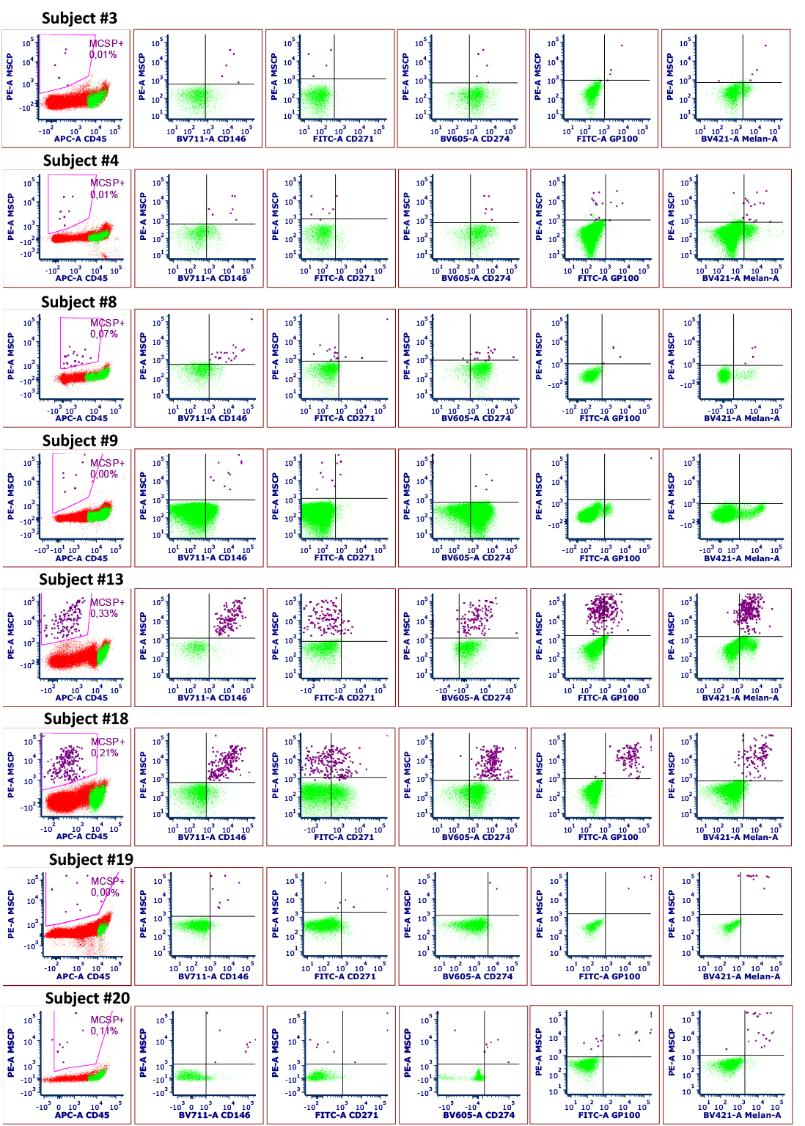
**

**Figure S3.** Digital droplet PCR for *BRAF* V600K mutation on sorted cells from subject 13 using fluorescence-activated cell sorting (FACS) **A**. Digital droplet PCR (ddPCR) on sorted lymphocytes, CD45+/CD146-/MCSP- cells **B.** ddPCR on sorted melanoma CTCs, CD45-/CD146+/MCSP+ cells. FACS; Fluorescence activated cell sorting

**
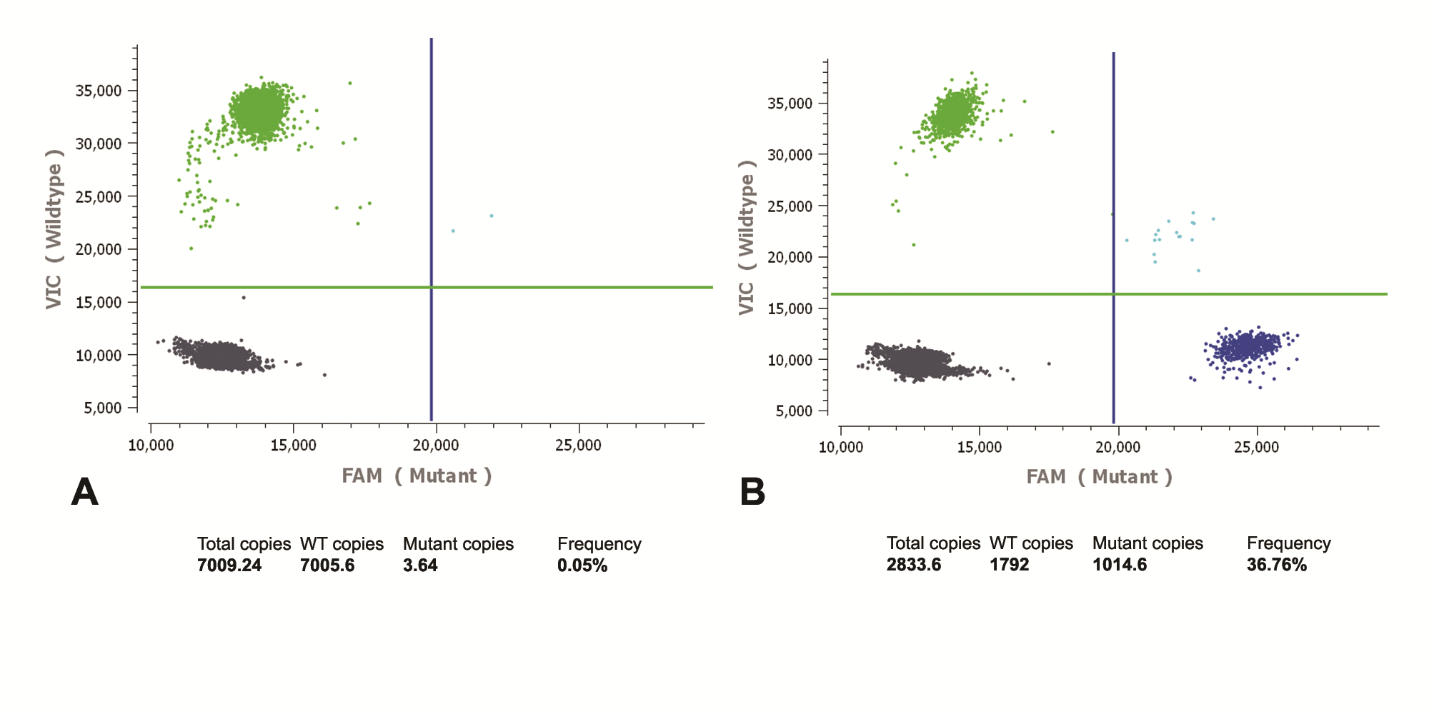
**

**Figure S4.** Correlation of cell-free DNA (cfDNA) measurement platforms. **A.** Correlation between variant allele frequency (VAF) and aneuploidy score **B.** Correlation between VAF and melanoma-specific methylation score **C.** Correlation between aneuploidy score and melanoma-specific methylation score


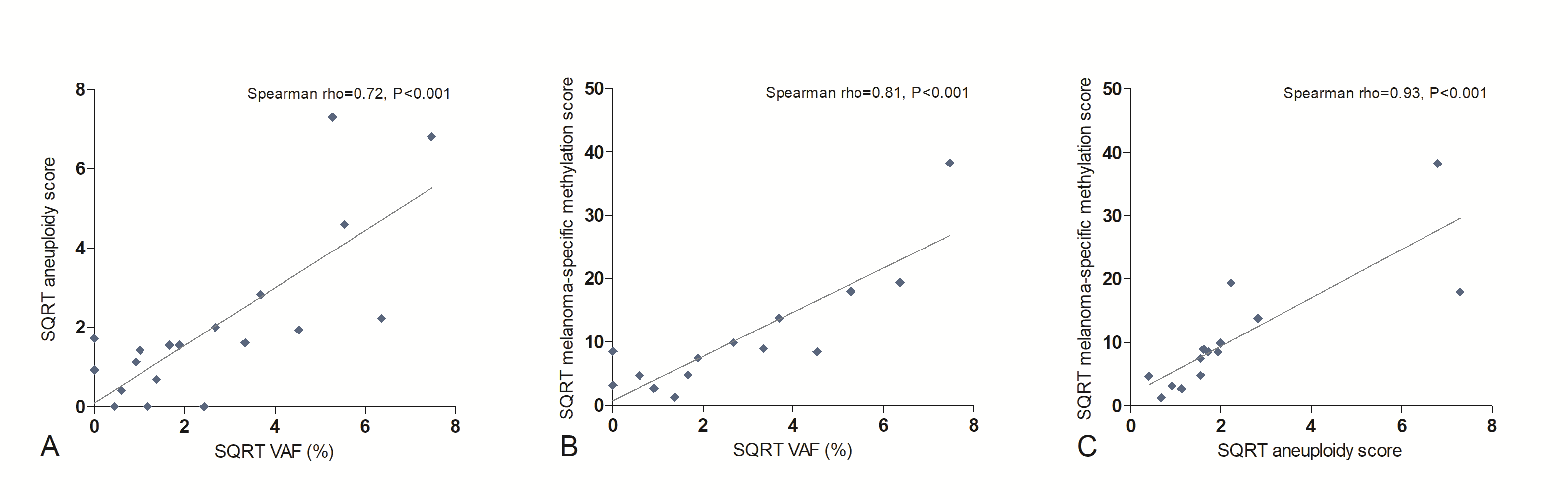


**Figure S5.** Principal component analysis of MeD-seq cfDNA methylation profiles from healthy blood donors (HBDs) and patients included in the study. PC1, PC2 and PC3 are shown on the x-axis, y-axis and z-axis, respectively. **Left:** samples are colored based on the presence of CTCs (HBDs in green, CTC count ≥1 in red and CTC count <1 in black). **Right**, samples are colored based on VAF (HBDs in green, VAF ≥1 in red and VAF <1 in black.


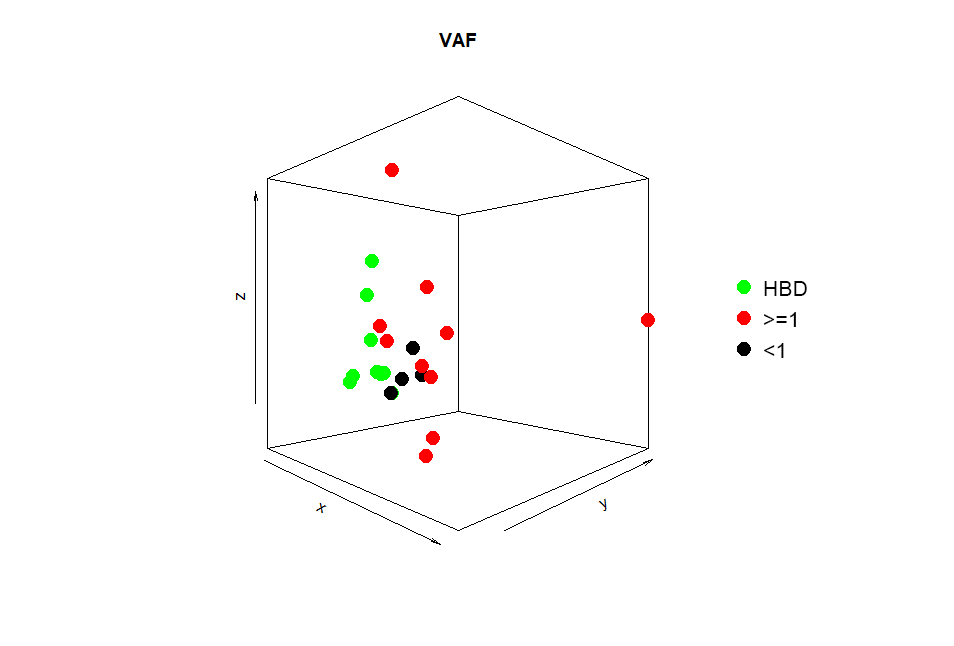

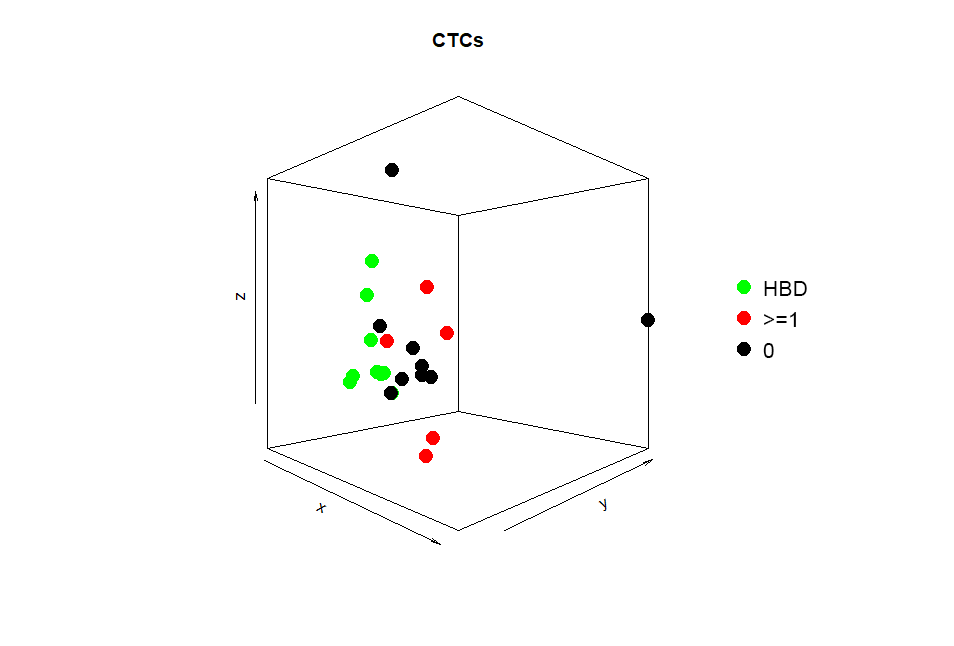


**Table S1.** Multi-color staining panel, using two tubes for membrane and intracellular markers

| **Component** | **Fluorochrome** | **Company (Clone)** | **Volumes (μL)** | |
| --- | --- | --- | --- | --- |
|  |  |  | **Tube 1 Membrane** | **Tube 2 Intracellular** |
| Cells suspension (~50µL) |  |  | Unfixed | Fixed with FIX&PERM® Kit, Caltag Medsystems, Buckingham, UK |
| Phosphate-Buffered Saline |  |  | 57 | - |
| Perm Solution |  | FIX&PERM® Kit, Caltag Medsystems, Buckingham, UK | - | 67 |
| DRAQ5, 250x diluted |  | BioStatus (DR50200) | 10 | 10 |
| HMW-MAA (MCSP) | PE | Menarini (CellSearch CMC kit staining Reagent) | 15 | 15 |
| CD45 and CD34 | APC | Menarini (CellSearch CMC kit staining Reagent) |  |  |
| CD274 20x diluted | BV605 | BD Biosciences  (740426, clone MIH1) | 5 | 5 |
| CD146 | BV711 | BD Biosciences  (563186, clone P1H12) | 1 | 1 |
| CD271 | BB15 (FITC) | BD Biosciences  (564580, clone C40-1457) | 2 | - |
| DAPI |  | Menarini CellSearch kit (Lot SLCK3406) | 10 | - |
| Melanoma Marker / GP100 | Alexa488 (FITC) | Bio-Connect (SC59305AF488, clone HMB45) | - | 1 |
| Melan-A/MART-1 | AF405 (BV405) | Novus  (NBP2-33148AF) | - | 1 |

**Table S2.** Overview of melanoma markers in all subjects. DRAQ5+/CD45-/MCAM+/MCSP+ cells were taken as reference (see also fig S2), as true melanoma origin was confirmed of these cells by genetic analysis after Flow cytometry analysis (see also Fig S1) NA: Not assessed

|  | **Membrane staining** | | | **Intracellular staining** | | |
| --- | --- | --- | --- | --- | --- | --- |
| **Subject** | **DRAQ5+/CD45-**  **MCAM+/MCSP+** | | | **DRAQ5+/CD45-**  **MCAM+/MCSP+** | | |
|  | **Total** | **CD271 +** | **CD274 +** | **Total** | **GP100 +** | **Melan-A +** |
| **1** | 3 | 3 (100%) | NA | 1 | 1 (100%) | NA |
| **2** | 0 | 0 | 0 | 0 | 0 | NA |
| **3** | 6 | 1 (17%) | 2 (33%) | 2 | 2 (100%) | 2 (100%) |
| **4** | 7 | 2 (29%) | 2 (29%) | 8 | 2 (25%) | 6 (75%) |
| **5** | 2 | 0 (0%) | 2 (100%) | 2 | 1 (50%) | 1 (50%) |
| **6** | 3 | 1 (33%) | 1 (33%) | 2 | 1 (50%) | 1 (50%) |
| **7** | 0 | 0 | 0 | 1 | 1 (100%) | 1 (100%) |
| **8** | 10 | 4 (40%) | 2 (20%) | 0 | 0 | 0 |
| **9** | 10 | 0 (0%) | 5 (50%) | 6 | 6 (100%) | 5 (83%) |
| **10** | 0 | 0 | 0 | 0 | 0 | 0 |
| **11** | 0 | 0 | 0 | 2 | 2 (100%) | 2 (100%) |
| **12** | 0 | 0 | 0 | 0 | 0 | 0 |
| **13** | 122 | 28 (23%) | 53 (43%) | 145 | 118 (81%) | 141 (97%) |
| **14** | 10 | 4 (40%) | 8 (80%) | 2 | 0 | 2 (100%) |
| **15** | 0 | 0 | 0 | 0 | 0 | 0 |
| **16** | 0 | 0 | 0 | 6 | 6 (100%) | 6 (100%) |
| **17** | 0 | 0 | 0 | 0 | 0 | 0 |
| **18** | 217 | 106 (49%) | 171 (78%) | 260 | 257 (99%) | 243 (93%) |
| **19** | 11 | 7 (64%) | 2 (18%) | 14 | 14 (100%) | 13 (93%) |
| **20** | 11 | 6 (55%) | 5 (45%) | 14 | 10 (71%) | 11 (79%) |

**Table S3.** List with 118 differentially methylated regions in patients with a mFastSeqS z-score ≥3 and FDR<0.1

| **Unique ID** | **FDR** |
| --- | --- |
| 7_66901857_66903857 | 0.012 |
| 15_96341953_96343953 | 0.022 |
| 8_76680219_76682219 | 0.022 |
| 7_137846092_137848092 | 0.022 |
| 7_27168564_27170564 | 0.022 |
| 7_51315818_51317818 | 0.022 |
| 15_20343736_20345736 | 0.022 |
| 1_167629674_167631674 | 0.022 |
| 20_50690528_50692528 | 0.022 |
| 15_76335724_76337724 | 0.031 |
| 4_84687000_84689000 | 0.034 |
| 9_136686423_136688423 | 0.034 |
| 14_24170853_24172853 | 0.035 |
| 8_53875649_53877649 | 0.036 |
| 7_108455717_108457717 | 0.036 |
| 12_53964965_53966965 | 0.036 |
| 20_64076010_64078010 | 0.038 |
| 1_161037962_161039962 | 0.050 |
| 1_161037990_161039990 | 0.050 |
| 5_7395208_7397208 | 0.062 |
| 22_18857640_18859640 | 0.064 |
| 6_167767568_167769568 | 0.064 |
| 1_92216940_92218940 | 0.064 |
| 12_48997367_48999367 | 0.064 |
| 3_24494282_24496282 | 0.064 |
| 8_143016982_143018982 | 0.064 |
| 17_61451404_61453404 | 0.064 |
| 7_157005307_157007307 | 0.064 |
| 2_230995121_230997121 | 0.067 |
| 1_47231220_47233220 | 0.067 |
| 7_129784185_129786185 | 0.067 |
| 2_219252243_219254243 | 0.071 |
| 19_38303161_38305161 | 0.073 |
| 14_103122007_103124007 | 0.073 |
| 12_118245084_118247084 | 0.084 |
| 16_67527675_67529675 | 0.085 |
| 16_22164583_22166583 | 0.086 |
| 2_230995032_230997032 | 0.086 |
| 10_98394307_98396307 | 0.086 |
| 2_94759279_94761279 | 0.086 |
| 1_120196085_120198085 | 0.086 |
| 12_49089208_49091208 | 0.086 |
| 15_20007432_20009432 | 0.086 |
| 20_34310802_34312802 | 0.086 |
| 7_5427731_5429731 | 0.086 |
| 7_5427927_5429927 | 0.086 |
| 14_64703102_64705102 | 0.086 |
| 1_38088231_38090231 | 0.089 |
| 15_21014078_21016078 | 0.089 |
| 5_1593620_1595620 | 0.089 |
| 5_1593626_1595626 | 0.089 |
| 2_27077567_27079567 | 0.089 |
| 7_27167619_27169619 | 0.089 |
| 22_30265840_30267840 | 0.089 |
| 6_167678270_167680270 | 0.090 |
| 17_80941033_80943033 | 0.092 |
| 9_134504472_134506472 | 0.092 |
| 1_201030136_201032136 | 0.092 |
| 19_2728327_2730327 | 0.092 |
| 5_173413035_173415035 | 0.092 |
| 15_20939252_20941252 | 0.092 |
| 6_133891802_133893802 | 0.092 |
| 3_155744067_155746067 | 0.092 |
| 5_96934809_96936809 | 0.092 |
| 22_50547033_50549033 | 0.092 |
| 5_96934394_96936394 | 0.092 |
| 2_165793682_165795682 | 0.092 |
| 12_10504602_10506602 | 0.092 |
| 16_9443985_9445985 | 0.092 |
| 17_81033881_81035881 | 0.092 |
| 14_59464342_59466342 | 0.092 |
| 14_91416844_91418844 | 0.092 |
| 1_143634641_143636641 | 0.092 |
| 4_188159065_188161065 | 0.092 |
| 8_81630134_81632134 | 0.092 |
| 15_62065977_62067977 | 0.092 |
| 2_161423332_161425332 | 0.092 |
| 2_161422577_161424577 | 0.092 |
| 2_161423015_161425015 | 0.092 |
| 2_44939045_44941045 | 0.092 |
| 21_25560909_25562909 | 0.092 |
| 14_94917734_94919734 | 0.092 |
| 6_1384066_1386066 | 0.092 |
| 11_64339223_64341223 | 0.092 |
| 11_59490652_59492652 | 0.092 |
| 12_47078951_47080951 | 0.092 |
| 1_161068970_161070970 | 0.092 |
| 14_36657801_36659801 | 0.092 |
| 1_148807181_148809181 | 0.092 |
| 11_131203999_131205999 | 0.092 |
| 6_27132042_27134042 | 0.092 |
| 5_139258_141258 | 0.092 |
| 2_159903710_159905710 | 0.092 |
| 2_159903749_159905749 | 0.092 |
| 16_56634739_56636739 | 0.092 |
| 19_8777861_8779861 | 0.092 |
| 7_27101811_27103811 | 0.092 |
| 16_27065707_27067707 | 0.092 |
| 7_19143293_19145293 | 0.092 |
| 1_66751459_66753459 | 0.092 |
| 4_985997_987997 | 0.092 |
| 7_27192448_27194448 | 0.092 |
| 2_240455001_240457001 | 0.092 |
| 9_119934053_119936053 | 0.092 |
| 7_27187816_27189816 | 0.092 |
| 20_63729377_63731377 | 0.092 |
| 5_70461244_70463244 | 0.092 |
| 5_163415525_163417525 | 0.093 |
| 16_29745006_29747006 | 0.094 |
| 17_78129770_78131770 | 0.094 |
| 22_23762021_23764021 | 0.095 |
| 2_104866496_104868496 | 0.096 |
| 22_30921308_30923308 | 0.096 |
| 1_146376184_146378184 | 0.097 |
| 6_149963007_149965007 | 0.097 |
| 21_44438035_44440035 | 0.098 |
| 13_18723904_18725904 | 0.099 |
| 7_39832793_39834793 | 0.099 |

**Table S4.** Variant allele frequency, aneuploidy score and melanoma-specific methylation score measurements for each individual patient

| **Subject** | **Variant allele frequency (%)** | **Aneuploidy score** | **Melanoma-specific methylation score** |
| --- | --- | --- | --- |
| 1 | 20,55 | 3,75 | 71,7 |
| 2 | 2,79 | 2,39 | 23,0 |
| 3 | 1,03 | 2,00 | Failed |
| 4 | 40,5 | 4,96 | 375,3 |
| 5 | 11,15 | 2,59 | 79,8 |
| 6 | 55,8 | 46,37 | 1464,6 |
| 7 | 0,85 | 1,27 | 7,1 |
| 8 | 3,56 | 2,39 | 55,4 |
| 9 | 27,83 | 53,30 | 323,0 |
| 10 | 0 | 0,85 | 10,0 |
| 11 | 0,36 | 0,17 | 21,8 |
| 12 | 7,2 | 3,97 | 97,6 |
| 13 | 13,6 | 7,95 | 190,3 |
| 14 | 1,9 | 0,46 | 1,7 |
| 15 | 0 | 2,96 | 72,2 |
| 16 | 0,2 | -0,15 | Failed |
| 17 | 0,2 | -0,06 | Failed |
| 18 | 5,9 | -0,42 | Failed |
| 19 | 1,4 | -0,18 | Failed |
| 20 | 30,7 | 21,10 | Failed |
